# Stress related to wild canid predators near dairy sheep farms associated with increased somatic cell counts in bulk-tank milk

**DOI:** 10.1038/s41598-024-53887-3

**Published:** 2024-02-08

**Authors:** Eleni I. Katsarou, Neil Reid, Daphne T. Lianou, George C. Fthenakis

**Affiliations:** 1 Veterinary Faculty, University of Thessaly, 43100 Karditsa, Greece; 2 Institute for Global Food Security (IGFS), School of Biological Sciences, Queen’s University Belfast, Belfast, BT9 5DL UK

**Keywords:** Risk factors, Zoology, Animal behaviour, Animal physiology

## Abstract

We investigated the association between wild canid predators reported near sheep farms throughout Greece and somatic cell counts in bulk-tank milk as a reflection of milk quality. The study included 325 dairy sheep flocks, where bulk-tank milk somatic cell counts and total bacterial counts were measured and staphylococci were isolated. Farms were divided into three groups: Cohort A (farms with no reports of wild canid predators nearby), B (farms with canid predators (golden jackal and grey wolf) nearby yet with no experience of livestock losses to predation) and C (farms with canid predators nearby and livestock losses to predation). Somatic cell counts in bulk-tank milk of Cohort C farms were significantly higher, + 43% and + 29%, compared to those for Cohorts A and B, respectively: 0.617 × 10^6^ cells mL^−1^ versus 0.433 × 10^6^ or 0.477 × 10^6^ cells mL^−1^, respectively. The presence of wild canid predators near sheep farms was associated with lower quality milk potentially indicative of stress consistent with the potential effects of a landscape of fear. Increasing biosecurity measures at livestock farms, e.g., fencing, and presence of livestock guard dogs could minimise predation risk, whilst also improving livestock welfare by reducing predator-associated stress.

## Introduction

In ecology, so-called ‘landscapes of fear’ refer to a conceptual framework defining potential predation risk as perceived by prey. This may be defined as a behavioural characteristic of individual animals or animal populations and may depend upon the sensory modalities of the prey^1^. The most important factor influencing animals in the development of a ‘landscapes of fear’ is the (direct and perceived) predation risk^2,3^. Laundre et al.^2,3^ consider this as a spatial map of the animals’ cost of foraging^1^. In this respect, three factors may play a relevant role: (1) the diversity of the predator community, (2) the activity of predators and the intensity of predation and (3) the possibility of animals to predict the risk of an attack^4^. It is also notable that in such cases, attempts to avoid one predator might increase possibility of predation by another, a phenomenon termed as ‘risk enhancement’^5^.

Sheep production in Greece is the single most important component of the country’s agricultural business sector, generating 18% of the total income of the primary sector^6^. Sheep farming in Greece is characterized overwhelmingly by dairy production. After weaning of lambs, ewes are milked from 3 to 8 months, with milk sold for preparation of cheese or yoghurt. National sheep milk production is 716,000 tons annually^7^, exceeding cattle milk production (643,000 tons annually), a unique feature of Greece among European countries^8^.

Somatic cell counts present an important measure of milk quality and reflect the health status of the mammary gland by indicating the presence of intramammary infection in animals. Increased somatic cell counts in bulk-tank milk produced in sheep flocks in Greece can lead to a penalty in the purchase price of raw milk. Mastitis is the most important factor leading to increased somatic cell counts, at individual animal and at flock level. The bulk-tank milk can provide information about the level of intramammary infections within the flock. Bergonier and Berthelot^9^ indicate that somatic cell counts in the order of 0.65 × 10^6^ cells mL^−1^ in the bulk-tank milk would reflect a 15% prevalence of subclinical mastitis in ewes of the flock. Nevertheless, other factors may also lead to increased cell counts in bulk-tank milk. For example, recently, somatic cell counts in the bulk-tank milk have been found to increase at the start and the end of a lactation period^10^.

In the past, the first report on the effects of stress on milk quality was by Hinks^11^, who recorded an incident in which bombing caused a substantial modification on the composition of cow milk, whilst later Ling^12^ reported that worrying of cows by dogs caused fluctuations in the composition of their milk. Booth^13^ was the first to suggest that stressful ‘adverse environmental stimuli’ might lead to increased somatic cell counts in cows; thereafter, Wegner et al.^14^ provided experimental evidence that stressing conditions in the farm environment may lead to increased somatic cell counts in the absence of infection. This could be important in cattle, as adverse environmental conditions, e.g., increased heat and/or relative humidity, have been found to contribute to higher somatic cell counts in the milk of these animals^15,16^. Other stressing factors might also affect cell counts; these include transportation of animals^17^ and vibration of the milking system at the time of milking^18^. In cows, thermal stress was recognised to lead to increased somatic cell counts^19,20^ and animal transportation was reported as well^17^. Stress conditions could act via the hypothalamic–pituitary–adrenal axis and could elicit various responses, including increased blood leucocyte numbers^14^, which then would lead to high somatic cell counts in milk even in the absence of mammary infection^14,21^.

In general, there is little information about the effect of stressing conditions on somatic cell counts of milk of ewes. The potential significance of the presence of wild canids that can predate sheep, near the flocks has never been reported and, unfortunately, in a recent review of stress-causing factors in sheep, wild predators have not been included and discussed^22^. Nevertheless, sheep are exposed to predation by wildlife mammals and the factor should be taken into consideration.

This study, carried out, as part of a large countrywide mapping of the sheep industry in Greece, aimed to assess variation in somatic cell counts in bulk-tank milk of sheep flocks in relation to the presence and potential impact of wild canid predators, namely, grey wolf (*Canis lupus*) and golden jackal (*Canis aureus*), which are responsible for livestock losses. The specific objectives of the study were to survey perceived wild canid predator occurrence near farms and losses of livestock to predation annually and to evaluate relevant associations with somatic cell counts in the bulk-tank milk of the farms. Our hypothesis was that presence of wild canids near the farms potentially increases somatic cell counts in bulk-milk, due to a landscape of fear effect, thus leading to reduced milk quality.

## Results

Wild canid predators were reported near 179 farms (55.1%), with grey wolf at 129 farms (39.7%) and golden jackal at 65 farms (20.0%). Livestock losses due to wild canid predators were reported at 74 farms (41.3%); predation by grey wolf was reported at 56 farms and by golden jackal at 19 farms.

**Figure 1:**
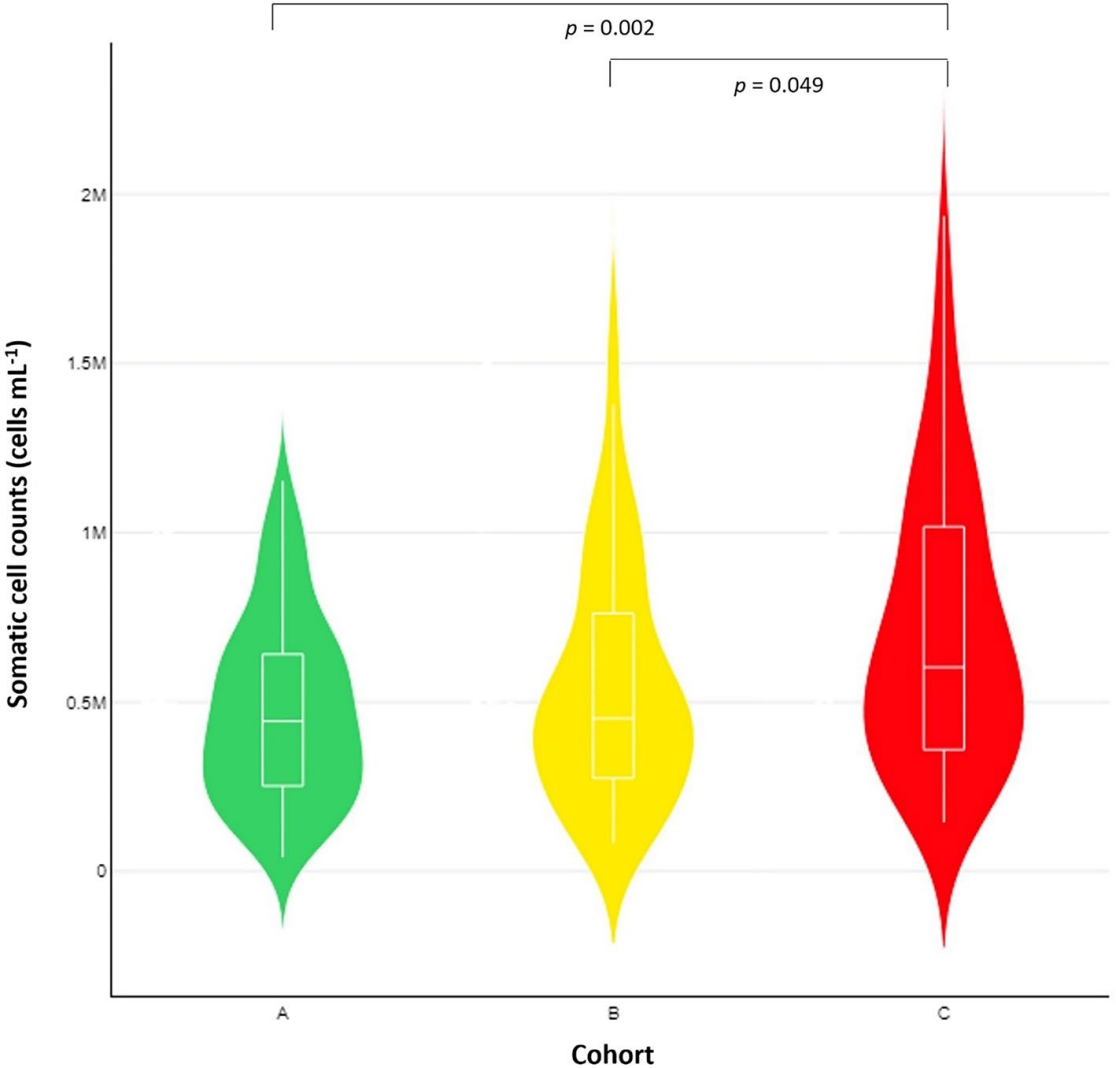
Somatic cell counts in bulk-tank milk among 325 sheep flocks, in accord with presence of predators nearby (cohorts B and C) and occurrence of livestock losses by predators (cohort C).

Mean somatic cell counts in bulk-tank milk of Cohort C farms were 42.5% higher than in Cohort A and 29.4% higher than in Cohort B: 0.617 × 10^6^ cells mL^−1^ versus 0.433 × 10^6^ cells mL^−1^ and 0.477 × 10^6^ cells mL^−1^, respectively (*p* = 0.003 (analysis of variance_df=2,322_); pairwise comparisons were as follows: Cohort C versus Cohort A *p* = 0.002, Cohort C versus Cohort B *p* = 0.049 and Cohort A versus Cohort B *p* > 0.050 (Tukey HSD)) (Table 1, Fig. 1). During the multivariable analysis_df=6,318_ (*F* = 5.228, *p* < 0.0001), significant associations of somatic cell counts were found with the following variables: (a) presence of wild canid predators near the farms (*p* = 0.005), (b) the month of the lactation period at sampling (*p* = 0.028) and (c) the education level of the farmer (*p* = 0.044).

**Table 1:** Somatic cell counts and microbiological parameters in bulk-tank milk among 325 sheep flocks.

Cohort^1^	Somatic cell counts (cells mL^−1^)^2^	Proportion of farms with SCC ≥ 0.650 × 10^6^ cells mL^−1^	Total bacterial counts (c.f.u. mL^−1^) ^2^	Proportion of farms with staphylococcal recovery
A (*n* = 146)	0.433 × 10^6^ (0.385 × 10^6^–0.487 × 10^6^)^a^	28.8%	378 × 10^3^ (295 × 10^3^–490 × 10^3^)	63.7%
B (*n* = 105)	0.477 × 10^6^ (0.415 × 10^6^–0.552 × 10^6^)^b^	34.3%	412 × 10^3^ (295 × 10^3^–575 × 10^3^)	63.8%
C (*n* = 74)	0.617 × 10^6^ (0.537 × 10^6^–0.708 × 10^6^)^a,b^	48.6%	418 × 10^3^ (288 × 10^3^–603 × 10^3^)	64.9%
*p*-value	0.003^3^	0.014^4^	0.880^3^	0.980^4^

^1^Cohort A: neither presence of predators nearby, nor occurrence of livestock losses by predators, cohort B: presence of predators nearby with no occurrence of livestock losses by these, cohort C: presence of predators nearby and occurrence of livestock losses by predators.

^2^Mean (95% confidence intervals).

^3^Analysis of variance_df=2,322_.

^4^χ^2^_df=2_.

^a,b^Significant difference (*p* < 0.05) within the same column between values in rows with the same superscript (Tukey HSD).

The proportion of farms with somatic cell counts in bulk-tank milk ≥ 0.650 × 10^6^ cells mL^−1^ was significantly higher within Cohort C farms than within Cohort A or Cohort B ones: 48.6% versus 28.8% and 34.3%, respectively (*p* = 0.014 (χ^2^_df=2_)). There were no differences between the three cohorts in the microbiological parameters assessed (*p* ≥ 0.850 for all comparisons (analysis of variance_df=2,322_ and χ^2^_df=2_)). Details are in Table 1.

Within Cohorts A and B, total bacterial counts in the sub-groups with somatic cell counts ≥ 0.650 × 10^6^ cells mL^−1^ were significantly higher than in the sub-groups with somatic cell counts < 0.650 × 10^6^ cells mL^−1^ (*p* = 0.001 (analysis of variance_df=1,144_) and *p* = 0.002 (analysis of variance_df=1,103_), respectively). In contrast, no such significant difference was seen within Cohort C (*p* = 0.240 (analysis of variance_df=1,72_). When the sub-groups with high or low somatic cell counts were compared between the three cohorts, no significant difference was found in total bacterial counts (*p* = 0.370 (analysis of variance_df=2,111_) between sub-groups with high somatic cell counts; *p* = 0.960 (analysis of variance_df=2,208_) between sub-groups with low somatic cell counts). Details are in Table 2. Further, a significant correlation was observed between somatic cell counts and total bacterial counts (*r* = 0.276, *p* = 0.0007 (Pearson correlation analysis_df=144_) and *r* = 0.325, *p* = 0.0007 (Pearson correlation analysis_df=103_), respectively) within Cohorts A and B. Such a significant correlation was not seen between these two parameters within Cohort C (*r* = 0.173, *p* = 0.14 (Pearson correlation analysis_df=72_)).

**Table 2:** Total bacterial counts and staphylococcal recovery from bulk-tank milk, according to somatic cell counts therein.

Cohort^2^	Total bacterial counts (c.f.u. mL^−1^)^1^			Proportion of farms with staphylococcal recovery		
	Farms with low cell counts^3^	Farms with high cell counts^3^	*p*^4^	Farms with low cell counts^3^ (%)	Farms with high cell counts^3^ (%)	*p*^5^
A (*n*^6^ = 104/42)	289 × 10^3^ (214 × 10^3^—398 × 10^3^)	729 × 10^3^ (490 × 10^3^–1096 × 10^3^)	0.001	60.6	71.4	0.210
B (*n* = 69/36)	286 × 10^3^ (200 × 10^3^—407 × 10^3^)	834 × 10^3^ (447 × 10^3^–1549 × 10^3^)	0.002	59.4	72.2	0.200
C (*n* = 38/36)	313 × 10^3^ (178 × 10^3^—550 × 10^3^)	566 × 10^3^ (347 × 10^3^–912 × 10^3^)	0.120	63.2	66.7	0.530

^1^Mean (95% confidence intervals).

^2^Cohort A: neither presence of predators nearby, nor occurrence of livestock losses by predators, cohort B: presence of predators nearby with no occurrence of livestock losses by these, cohort C: presence of predators nearby and occurrence of livestock losses by predators.

^3^Low cell counts: < 0.650 × 10^6^ cells mL^−1^, high cell counts: ≥ 0.650 × 10^6^ cells mL^−1^.

^4^Analysis of variance (_df=1,144_), (_df=1,103_), (_df=1,72_), for Cohorts A, B, C, respectively.

^5^χ^2^_df=2_.

^6^n_1_/n_2_ = number of farms within each cohort with somatic cell counts < 0.650 × 10^6^/ ≥ 0.650 × 10^6^, respectively.

Moreover, a significant correlation was observed between somatic cell counts and total bacterial counts (*r* = 0.276, *p* = 0.0007 (Pearson correlation analysis_df=144_) and *r* = 0.325, *p* = 0.0007 (Pearson correlation analysis_df=103_), respectively) within Cohorts A and B. Such a significant correlation between these two parameters was not seen within Cohort C (*r* = 0.173, *p* = 0.140 (Pearson correlation analysis_df=72_)).

Within Cohort C, there was no difference in the somatic cell counts of farms, in which sheep losses were caused by wolf or jackal: 0.638 × 10^6^ (95% CI 0.544 × 10^6^–0.749 × 10^6^) *versus* 0.552 × 10^6^ cells (95% CI 0.418 × 10^6^–0.728 × 10^6^) mL^−1^, respectively (*p* = 0.370 (analysis of variance_df=1,73_).Within the same cohort, there was a significant inverse correlation between the annual frequency of attacks by wild canid predators per farm and the somatic cell counts in the bulk-tank milk (*r*_*sp*_ = − 0.279, *p* = 0.016 (Spearman’s rank correlation analysis_df=72_)).

## Discussion

Exposure of animals to predators or predator cues can induce 'sustained psychological stress'^23^. Predation attacks to sheep lead to increased blood-cortisol concentration^24^, which are associated with increased somatic cell counts in the absence of mammary infection^25^. This can potentially explain the increased cell counts in the flocks where predation attacks were recorded.

The lack of differences in microbiological parameters (total bacterial counts and rate of staphylococcal recovery) between the two sub-groups within Cohort C lends support to a hypothesis that, at least to some extent, high somatic cell counts can be triggered as potential effects of sheep fear of the presence of canid predators (and risk of attack). The contrasting emergence of significance between respective sub-groups in Cohorts A and B and the lack of correlation between somatic cell counts and microbiological parameters in Cohort C (again in contrast to A and B) lend further support to this hypothesis.

The possibility that the increased somatic cell counts are the consequence of a higher incidence of mastitis in farms within Cohort C cannot be ruled out entirely. In such a scenario, mastitis in animals of farms within that cohort can be a consequence of stress to the immune system. In farm animals, pain, fear or the inability to perform a defined behavioural pattern are considered to lead to stress^26^. In turn, this exerts an enhanced secretion of glucocorticoids, which play a role in the down-regulation of interferon-*γ* and various pro-inflammatory cytokines (e.g., interleucin-1, inteleucin-2, granulocyte–macrophage colony-stimulating factor)^27^, which all participate in the defence response of the mammary gland during bacterial invasion.

The results point out that the potential effects of stress in milk somatic cell counts, as the result of presence of canid predators, can be as significant as those associated with established management-related variables. The start of the milking procedure is a confirmed predisposing factor for the development of mastitis in dairy ewes^28,29^, which explains the increased somatic cell counts at that period. At the end of the milking period, there is also an increase in somatic cell counts, that can occur also in the absence of infection^30^. Hence, those present findings are associated to previous results. However, the identification of stress-associated with the presence of canid predators near the farms is a novel and interesting finding.

Nevertheless, the repeated exposure to predation events is associated with lower cell counts, which may be accounted as attentional and judgment bias, due to learning deficits. This has been reported before to occur in sheep in cases of repeated and chronic stressing factors^31,32^. Responses to acute stressors are well documented, but chronic stress remains difficult to assess in farm animals and animal reactions may be difficult to predict^30^. Chronic stress can influence judgement and attention, as well as the activity of the hypothalamic–pituitary–adrenal axis in sheep^32^. Indeed, Verbeek et al.^32^ have reported that sheep exposed to chronic stress showed a slightly higher ‘optimism’ in their response compared to animals exposed to acute stress. Further, Boissy et al.^33^ have reported that calves exposed to novel or sudden events and to predator cues responded with a delay or with a milder reaction at the repeat performance of the annoying stimuli.

In cases of increased somatic cell counts, dairy companies often apply a penalty and consequently reduce the purchase price of the milk. The current findings indicate that the presence of predating wild canids near a farm may also contribute to increased somatic cell counts in milk, as a behavioural result of the population in the flock in front of predation stimuli and this can have adverse financial consequences for the farmers. It is also noteworthy that there is a widespread and undeniable impact to the welfare of livestock from wild predators^34^. This is something to take into consideration during assessment of small ruminant farms for the level of welfare applied therein.

Top predators are important for ecosystems as they regulate species at lower trophic levels within the food web (through so-called ‘trophic cascades’). Problems caused by livestock losses create human-wildlife conflict; in this case, specifically with shepherds who may action, for example, hunting to minimise predation risk from wild canids. Increasing farm biosecurity, for example, installation of fencing, housing livestock at night etc., can minimise risk, while livestock dogs are effective in deterring attacks. Within the context of the present study, creation of conditions to improve livestock ‘safety’ can also reduce their ‘fear’ and thus reduce stress relevant to milk quality.

## Methods

**Figure 2:**
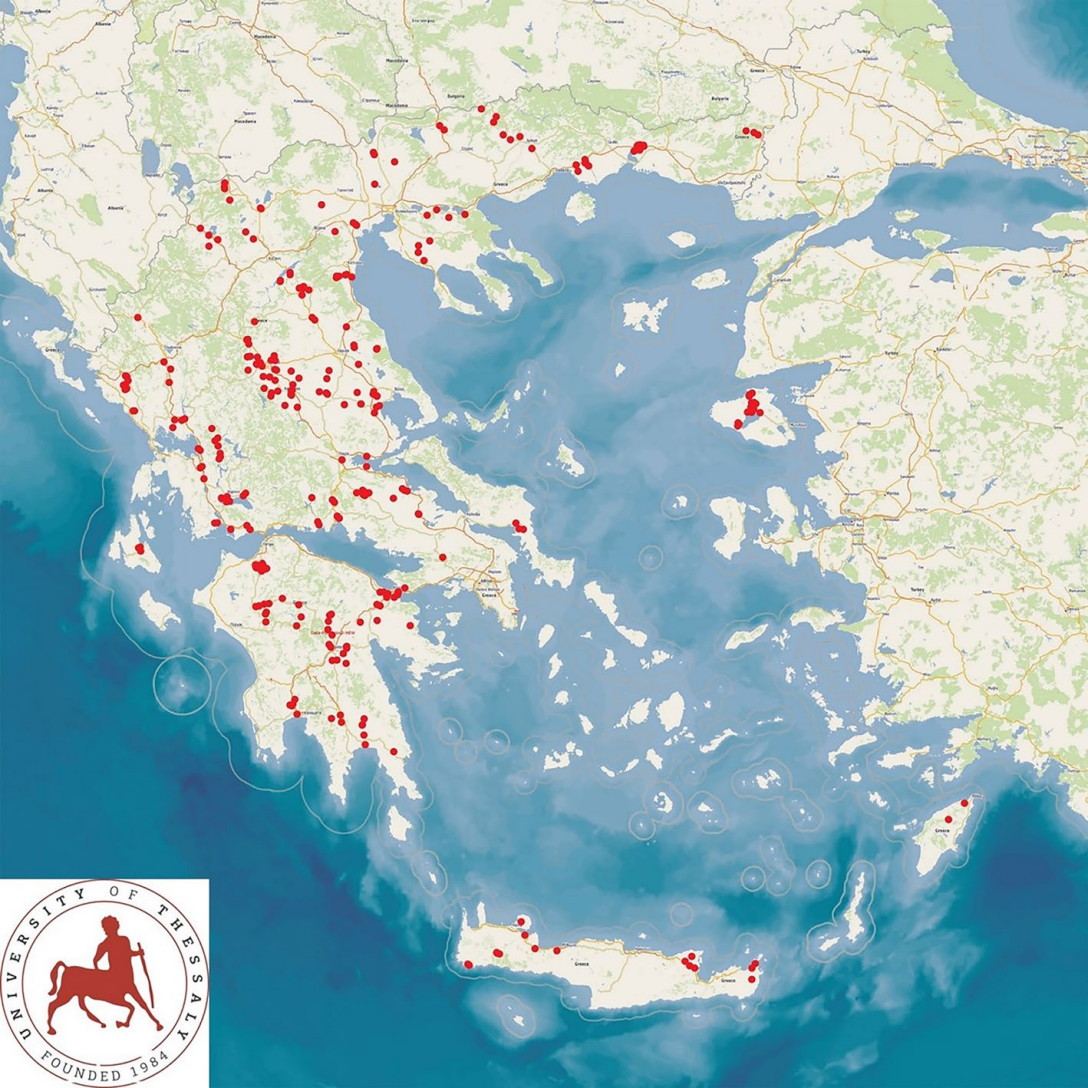
Location of the 325 sheep flocks around Greece, which were visited for collection of samples and information regarding somatic cell counts in milk and presence of canid wildlife predators near the farms (map drawn by use of GPS Visualizer (https://www.gpsvisualizer.com; Adam Schneider, Portland, OR, USA).

A cross-sectional study was performed across all 13 administrative regions of Greece. As part of the study, the investigators visited 325 sheep flocks for collection of samples and information. The farms visited during the study, were located in all the 13 administrative regions of Greece (Fig. 2). The flocks were included in the study on a convenience basis (specifically, the willingness of farmers to participate in the study and receive a visit by university staff for interview and sample collection), as detailed before^35^. The interview was carried out always by the same researcher (author D.T.L.), using a structured detailed questionnaire^35^. During the interview with farmers, information was obtained regarding the presence of canid wildlife predators near the farms (Supplementary information 1).

During the visit to each farm, data on the farm’s location were collected using hand-held Garmin global positioning system units. The geo-references were resolved to the specific farm level.

During the visit to each farm, four samples of milk (volume of each sample: 20 mL) were collected, by permission of the farmer, from the bulk-tank milk of the farm. For the sampling, standard techniques, i.e., thorough mixing of bulk-tank content and use of single use plastic pipettes for milk collection, were employed. For transport, samples were stored at 0.0–4.0 °C using ice packs in portable refrigerators. During the visit to the farms.

Somatic cell counting (Lactoscan SCC; Milkotronic Ltd, Nova Zagora, Bulgaria) was performed in duplicate in each of two samples collected from the bulk-tank (i.e., from each of two samples collected, two sub-samples were processed for cell counting). Testing was performed within 4 h of sampling.

Bacteriological examinations started within 24 h after collection of samples. These included total bacterial counting in the milk samples, following the procedure described by Laird et al.^36^, and culturing for recovery and identification of staphylococcal isolates, following the procedures, previously described in detail by Lianou et al.^10^. Again, from each of two samples collected, two sub-samples were processed for bacteriological examination.

For the evaluation of differences between farms, the farms were divided into three groups: Cohort A included sheep farms near which no canid wildlife predators (grey wolf, golden jackal) were reported, Cohort B included sheep farms near which canid wildlife predators were reported but sheep losses had not been noted in there, and Cohort C included sheep farms near which canid wildlife predators were reported and also sheep losses by predation to the wildlife species had occurred in there.

Subsequently, within each cohort, two sub-groups were created: one that included flocks with somatic cell counts in bulk-tank milk < 0.650 × 10^6^ cells mL^−1^ and one that included flocks with somatic cell counts in bulk-tank milk ≥ 0.650 × 10^6^ cells mL^−1^ (the value of 0.650 × 10^6^ cells mL^−1^ was indicated by Bergonier and Berthelot^9^ to correspond to 15% prevalence of subclinical mastitis in a flock).

For the statistical analysis, somatic cell counts were transformed as previously detailed^37,38^: somatic cell scores = log_2_(somatic cell counts/100) + 3; moreover, total bacterial counts were transformed to log_10_. The transformed data were used in the analyses; back-transformation of the results obtained was carried out for the presentation of the results.

Data were entered into Microsoft Excel and analyzed using SPSS v. 27 (IBM Analytics, Armonk, NY, USA). Initially, basic descriptive analysis was performed. Comparisons between somatic cell scores among the three cohorts were performed by using analysis of variance. Subsequently, a retrospective analysis was carried out, with the addition of management-related variables (*n* = 6) previously found with a significant association with somatic cell counts of bulk-tank milk in these farms^10^ (Supplementary information 2). A multivariable model was created, with the presence of predators near the farms and the above management-related variables. Variables were removed from the initial model by backwards elimination. The *p* value of removal of a variable was assessed by the likelihood ratio test, and for those with a *p* value of > 0.2 the variable with the largest probability was removed. This process was repeated until no variable could be removed with a *p* value of > 0.2. The variables required for the final multivariable test are shown in Supplementary information 3.

Comparisons between frequencies were performed by using Pearson’s chi-square test. Pearson correlation analysis was performed between somatic cell scores and log-transformed bacterial counts within the three groups. Finally, Spearman’ rank correlation analysis was used between the annual frequency of attacks of wild canids and the somatic cell counts. In all analyses, statistical significance was defined at *p* < 0.05.

During the study, all procedures, in the farms and in the laboratory, were performed in accordance with the relevant guidelines and regulations. The study has been reported in accordance with the ARRIVE guidelines.

### Ethics approval

The protocols of the study were approved by the academic board of the Veterinary Faculty of the University of Thessaly, meetings 34/03.04.19 and 82/04.11.20. The legal provisions regarding experimental procedures in Greece are determined by the provisions of Presidential decree 56/2013 (Official Gazette of the Government of the Hellenic Republic part A, issue 106, 30.04.13) and Ministerial decision 2416/83725/2016 (Official Gazette of the Government of the Hellenic Republic part B, issue 2323, 27.07.16), issued in accord with Directive 2010/63/ΕΕ of the European Parliament and the Council of 22nd September 2010 (Official Journal of the European Union L 276/33/20.10.2010).

### Supplementary Information

Supplementary Information.

## Data Availability

The dataset used and analyzed in the current study is available at http://upload.users.uth.gr/files/Katsarou-data.xlsx.
