# Supplementary material for: Stress related to wild canid predators near dairy sheep farms associated with increased somatic cell counts in bulk-tank milk
Source: Sci Rep. 2024 Feb 8;14:3252. doi: 10.1038/s41598-024-53887-3 (PMC10853181; doi:10.1038/s41598-024-53887-3)
Supplement: Supplementary file 1 — Supplementary Information. [file 41598_2024_53887_MOESM1_ESM.pdf]

Stress related to wild canid predators near dairy sheep farms associated with increased somatic cell counts in bulk-tank milk

Eleni I. Katsarou, Neil Reid, Daphne T. Lianou, George C. Fthenakis

Supplementary information 1.

Variables (*n* = 8) evaluated with regard to the presence of canid wildlife predators near a farm and the potential association with somatic cell counts in bulk-tank milk, during a countrywide study in 325 sheep flocks in Greece.

Visual contacts of the farmer with wildlife mammals (yes / no)
Wildlife mammals identified within a radius of 2 km of the farm (description)
Animal deaths from attacks by wildlife animals (yes / no)
Species of wildlife animals that caused animal deaths (description)
Animal deaths from attacks by wildlife animals (yes / no)
Species of wildlife animals that caused animal deaths during the preceding season (description)
No. of ewes / does in the flock / herd (no.)
Total cases of clinical mastitis during the preceding season (no.)

Supplementary information 2.

Variables (n = 6) with significant variations between their categories with regard to somatic cell counts (mean [95% confidence intervals] cells mL^{-1}) in bulk-tank milk, during a countrywide study in 325 sheep flocks in Greece [1].

**Month into the lactation period at sampling**				
0–1st (n = 23)	2nd–5th (n = 138)	6th–9th (n = 147)	After 9th (n = 17)	p
0.760×10^{6} (0.575×10^{6}–1.002×10^{6})	0.494×10^{6} (0.439×10^{6}–0.556×10^{6})	0.440×10^{6} (0.393×10^{6}–0.490×10^{6})	0.608×10^{6} (0.436×10^{6}–0.848×10^{6})	0.004

**Availability of milking parlour**		
Yes (n = 224)	No (n = 31)	p
0.474×10^{6} (0.433×10^{6}–0.518×10^{6})	0.358×10^{6} (0.260×10^{6}–0.461×10^{6})	0.034

**Month of the start of the lambing season**					
All year (n = 18)	Aug.–Sep. (n = 75)	Oct.–Nov. (n = 170)	Dec.–Jan. (n = 48)	Feb.–Jul. (n = 14)	p
0.554×10^{6} ($0.427\ 10^{6}$–0.713×10^{6})	0.477×10^{6} (0.407×10^{6}–0.560×10^{6})	0.440×10^{6} (0.393×10^{6}–0.490×10^{6})	0.672×10^{6} (0.548×10^{6}–0.819×10^{6})	0.566×10^{6} (0.407×10^{6}–0.791×10^{6})	0.006

**Annual incidence of clinical mastitis**		
≤ 0.50% (n = 56)	> 0.50% (n = 269)	p
0.411×10^{6} (0.357×10^{6}–0.477×10^{6})	0.506×10^{6} (0.208×10^{6}–1.242×10^{6})	0.049

**Age of lamb removal from their dams**			
< 45 days (n = 119)	45–60 days (n = 170)	> 60 days (n = 36)	p
0.423×10^{6} (0.374×10^{6}–0.480×10^{6})	0.525×10^{6} (0.474×10^{6}–0.583×10^{6})	0.558×10^{6} (0.424×10^{6}–0.738×10^{6})	0.020

**Highest level of education of farmer**		
Primary or secondary (n = 222)	Over secondary (n = 103)	p
0.519×10^{6} (0.474×10^{6}–0.567×10^{6})	0.429×10^{6} (0.372×10^{6}–0.497×10^{6})	0.027

1. Lianou DT et al. (2021) Extensive countrywide field investigation of somatic cell counts and total bacterial counts in bulk-tank raw milk in sheep flocks in Greece. Foods 10:268.

Supplementary information 3.

Details of a multivariable model employed for the evaluation of potential association of the presence of wild canid predators near sheep farms with somatic cell counts in bulk-tank milk, during a countrywide study in 325 sheep flocks in Greece.

Outcome	Variables offered to the multivariable model	Variables required in the final test
Somatic cell counts in bulk-tank milk	7	(a) Presence of wild canid predators near the farms, (b) Month into the lactation period at sampling, (c) Availability of milking parlour, (d) Annual incidence of clinical mastitis, (e) Age of lamb removal from their dams, (f) Highest level of education of farmer
